# Differences in the stemness characteristics and molecular markers of distinct human oral tissue neural crest‐derived multilineage cells

**DOI:** 10.1111/cpr.13286

**Published:** 2022-06-18

**Authors:** Shigehiro Abe, Atsushi Kaida, Kazunori Kanemaru, Keiichiro Nakazato, Naoko Yokomizo, Yutaka Kobayashi, Masahiko Miura, Toshio Miki, Chiaki Hidai, Hisataka Kitano, Tetsuya Yoda

**Affiliations:** ^1^ Division of Oral Surgery, Faculty of Medicine Nihon University Itabashi‐ku, Tokyo Japan; ^2^ Department of Dentistry and Oral Surgery Tokyo Metropolitan Hiroo Hospital Shibuya‐ku, Tokyo Japan; ^3^ Department of Oral Radiation Oncology, Graduate School of Medical and Dental Sciences Tokyo Medical and Dental University Bunkyo‐ku, Tokyo Japan; ^4^ Department of Physiology, Graduate School of Medicine and Faculty of Medicine Nihon University Itabashi‐ku, Tokyo Japan; ^5^ Department of Maxillofacial Surgery, Graduate School of Medical and Dental Sciences Tokyo Medical and Dental University Bunkyo‐ku, Tokyo Japan

## Abstract

**Objectives:**

Although multilineage cells derived from oral tissues, especially the dental pulp, apical papilla, periodontal ligament, and oral mucosa, have neural crest‐derived stem cell (NCSC)‐like properties, the differences in the characteristics of these progenitor cell compartments remain unknown. The current study aimed to elucidate these differences.

**Material and methods:**

Sphere‐forming apical papilla‐derived cells (APDCs), periodontal ligament‐derived cells (PDLDCs), and oral mucosa stroma‐derived cells (OMSDCs) from the same individuals were isolated from impacted developing teeth. All sphere‐forming cells were characterized through biological analyses of stem cells.

**Results:**

All sphere‐forming cells expressed neural crest‐related markers. The expression of certain tissue‐specific markers such as CD24 and CD56 (NCAM1) differed among tissue‐derived cells. Surprisingly, the expression of only CD24 and CD56 could be discriminated in human tissues. Although APDCs and PDLDCs exhibited greater mineralized cell differentiation than OMSDCs, they exhibited poorer differentiation into adipocytes *in vitro*. In immunocompromised mice, APDCs formed hard tissues better than PDLDCs and OMSDCs.

**Conclusions:**

Although cells with NCSC‐like properties present the same phenotype, they differ in the expression of certain markers and differentiation abilities. This study is the first to demonstrate the differences in the differentiation ability and molecular markers among multilineage human APDCs, PDLDCs, and OMSDCs obtained from the same patients, and to identify tissue‐specific markers that distinguish tissues in the developing stage of the human tooth with immature apex.

## Graphical abstract

This study illustrates that neural crest‐derived cells from distinct oral tissues, namely the apical papilla, periodontal ligament, and oral mucosa, have varying differentiation potential, and tissue‐derived cell‐specific molecular markers have been identified. CD24 and NCAM1/CD56 expression were found to differ among multilineage oral tissue‐derived cells, similar to our observation in human tissue.

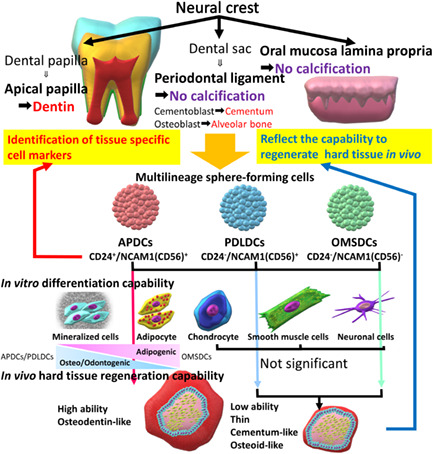

## 1 INTRODUCTION

Oral and maxillofacial tissues are attractive cell sources for regenerative medicine. A sufficient number of stem cells can be obtained from the third molar tissue without causing aesthetic issues or collecting surplus tissue. Dental pulp stem cells (DPSCs),^1^, ^2^, ^3^, ^4^, ^5^, ^6^ periodontal ligament stem cells (PDLSCs),^7^ and oral mucosa stromal stem cells (OMSCs)^8^, ^9^ are derived from the neural crest (NC).^10^, ^11^ NC‐derived stem cell (NCSC)‐like cells have been isolated from various mice and human tissues, including the skin,^12^, ^13^, ^14^ heart,^15^ bone marrow,^16^ dental pulp,^17^, ^18^, ^19^, ^20^ periodontal ligament,^21^, ^22^ and oral mucosa,^23^, ^24^ using the sphere formation technique, which can enrich stem/progenitor cells.^12^, ^13^, ^14^, ^15^, ^16^, ^17^, ^18^, ^19^, ^20^, ^21^, ^22^, ^23^, ^24^ NCSCs express NC‐related markers and can differentiate into the cells of mesenchymal lineage, including osteoblasts, chondrocytes, adipocytes, and smooth muscle cells, as well as cells of neural lineage.^25^

Although DPSCs are present in the dental pulp tissue of human teeth with completely formed roots, the apical papilla from human teeth with immature root apices is a developing tissue, and the stem cells present play a crucial role in complete root formation. Sonoyama *et al*. reported that stem cells from the apical papilla have higher proliferative potential, higher telomerase activity, and a greater capacity for hard/mineralized‐tissue formation than stem cells from DPSCs.^4^ Similarly, it is expected that the periodontal ligament from developing human teeth also contains stem cells capable of forming cementum and periodontal ligaments along with tooth roots. Therefore, we consider that developing human teeth are an attractive source of stem cells for regenerative medicine.^5^, ^6^, ^17^, ^18^

Another source of stem cells, the oral mucosa with a very high regenerative ability following injury, and can be obtained even from patients without teeth.^23^ Studies have shown that dental pulp, apical papilla, periodontal ligaments, and oral mucosa‐derived stem/progenitor cells have the characteristics of NCSCs, with each type of tissue‐derived cell considered to possess identical stem/progenitor cell properties;^17^, ^18^, ^19^, ^20^, ^21^, ^22^, ^23^, ^24^, ^26^ however, these differences have not been characterized. Elucidating these cell‐specific characteristics may help not only in demonstrating the importance of each cell type with respect to tissue regeneration via differentiation into suitable target lineages, but also in identifying tissue‐specific markers and their developmental role in the currently unknown human tooth developmental process.

The apical papilla, periodontal ligament, and oral mucosa can be obtained concomitantly during the extraction of an impacted third molar with an immature apex. To the best of our knowledge, differences in the differentiation ability and expression of molecular markers among human apical papilla‐derived cells (APDCs), periodontal ligament‐derived cells (PDLDCs), and oral mucosa stroma‐derived cells (OMSDCs) obtained from individuals simultaneously and from the same sites have not been reported previously. This study was performed to characterize human NCSC‐like cells from tissues obtained through the extraction of impacted developing third molars and to confirm the differences in cellular characteristics and molecular markers of individual tissue‐derived stem cells, which have a great clinical scope and are potentially valuable in dental regenerative medicine.

## 2 MATERIALS AND METHODS

Detailed materials and methods are described in the supplementary material (Extended Materials and Methods).

### 2.1 Patients

Human apical papilla, periodontal ligaments, and oral mucosa tissues were obtained for the extraction of impacted developing third molars. Written informed consent was obtained from all donors.

### 2.2 Histological analysis

Prepared sections were used for hematoxylin–eosin (H&E) and immunohistochemical staining for nestin, CD44, CD24, and CD56 (NCAM1).

### 2.3 Cell culture

APDCs, PDLDCs, and OMSDCs were cultured in Isocove's modified Dulbecco's medium (Nacalai Tesque, Kyoto, Japan) supplemented with 10% fetal bovine serum (FBS) (Gibco Life Technologies, Carlsbad, CA, USA, Lot number: 42A0158K). APDCs, PDLDCs, and OMSDCs were passaged and cryopreserved.^5^, ^6^, ^17^, ^18^, ^23^

Oral mucosal epithelial cells were cultured in keratinocyte SFM (Gibco Life Technologies). The human tongue squamous cell carcinoma cell line (SCCKN) was cultured in 45% Dulbecco's modified Eagle medium (DMEM) (Gibco Life Technologies), 45% RPMI 1640 medium (Gibco Life Technologies), and 10% FBS.

### 2.4 Growth of APDCs, PDLDCs, and OMSDCs

The growth assay was performed as previously described.^23^

### 2.5 Colony‐forming assay

APDCs, PDLDCs, and OMSDCs from five patients were seeded at a density of 500 cells/well in 6‐well plates. After 14 days of culture, the colonies were stained and counted as previously described.^23^

### 2.6 Sphere culture

APDCs, PDLDCs, and OMSDCs were cultured in 24‐well super‐hydrophilic plates (Cellseed, Tokyo, Japan) in serum‐free DMEM/F12 (1:1) containing N_2_ supplements (Gibco Life Technologies), 20 ng/ml basic fibroblast growth factor (PeproTech, Rocky Hill, NJ, USA), and 20 ng/ml epidermal growth factor (PeproTech) for seven days.^17^, ^18^, ^23^

### 2.7 Immunohistochemistry

Immunohistochemistry was performed as previously described.^23^ Slides were incubated at room temperature for 1 h with primary antibodies (see Extended Materials and Methods). The cells were washed twice with phosphate‐buffered saline (PBS) and incubated at room temperature for 30 min with Alexa Fluor 488–conjugated anti‐mouse IgG (Invitrogen, Carlsbad, CA, USA; 1:200) and Alexa 594– conjugated anti‐mouse or anti‐rat IgG (Invitrogen; 1:200).^23^

### 2.8 Flow cytometry

Sphere‐forming APDCs, PDLDCs, and OMSDCs were enzymatically dissociated and washed with PBS. The cells were incubated at room temperature for 1 h with primary antibodies (Extended Materials and Methods). After incubation, the cells were washed with PBS, then incubated with anti‐mouse or anti‐rat IgG conjugated with Alexa 488 or 594 (Invitrogen; 1:200) at room temperature for 30 min. Subsequently, the cells were analyzed with a FACSCant™ (BD Biosciences, Franklin Lakes, NJ, USA).

### 2.9 RNA extraction

Total RNA was extracted from the cells using the RNeasy Mini Kit (Qiagen, Hilden, Germany), according to manufacturer instructions.

### 2.10 Microarray

The total RNA of sphere‐forming APDCs, PDLCs, and OMSCs (100 ng) was reverse‐transcribed into cDNA using the Low Input Amp Labeling Kit (Agilent Technologies, Santa Clara, CA, USA). Afterward, cDNA was mixed with a hybridization buffer and hybridized to a SurePrint G3 Human GE microarray 8X60k v3 (Agilent Technologies) for 17 h by following manufacturer instructions. The chips were washed and subsequently scanned using the Agilent microarray scanner (Agilent Technologies). Raw data were analyzed using the feature extraction software (v.11.5.1.1; Agilent Technologies), and microarray data were mined and analyzed using GeneSpring (Agilent Technologies). The signal intensity was normalized by adjusting the data to a 75th percentile baseline (GEO accession number for the microarray gene expression data: GSE164327).

### 2.11 Semi‐quantitative and quantitative reverse transcription PCR (RT‐PCR)

Semi‐quantitative RT‐PCR was performed using the PrimeScript One‐Step RT‐PCR Kit Ver.2 (Takara, Kusatsu, Japan). Quantitative RT‐PCR was performed using the One‐Step PrimeScript RT‐PCR Kit (Perfect Real Time; Takara). The primer sequences used for RT‐PCR are listed in Table 1.^23^, ^27^, ^28^

**TABLE 1:** Primers used for RT‐PCR

Gene		Primer/Probe sequence (5′ to 3′)	GenBank	Size (bp)	Reference primer bank ID/Assay name
*NES*	F	CCTGGGAAAGGGAGA GTACC	NM_006617	111	27
	R	TGGTCCTTCTCCACCGTA TC			
*CD44*	F	CTGCCGCTTTGCAGGTGTA	NM_000610	109	28
	R	CATTGTGGGCAAGGTGCTATT			
*SNAI1*	F	TCGGAAGCCTAACTACAGCGA	NM_005985	140	301336132c1
	R	AGATGAGCATTGGCAGCGAG			
*SNAI2*	F	TGTGACAAGGAATATGTGAGCC	NM_003068	203	324072669c2
	R	TGAGCCCTCAGATTTGACCTG			
*MSX1*	F	CTCCGCAAACACAAGACGAAC	NM_002448	391	23
	R	CACATGGGCCGTGTAGAGTC			
*Hes1*	F	TCAACACGACACCGGATAAAC	NM_005524	153	325652058c1
	R	GCCGCGAGCTATCTTTCTTCA			
*GAPDH*	F	AGCAATGCCTCCTGCACCACCAAC	NM_002046	137	23
	R	CCGGAGGGGCCATCCACAGTCT			
*RUNX2*	F	AGGCGGTCAGAGAACAAAC	NM_001015051	129	Hs.PT.56a.19568141
	R	CTTCACAAATCCTCCCCAAGT			
*SP7*	F	GGAGCCATAGTGAACTTCCTC	NM_152860	94	Hs.PT.58.3437800
	R	AGCTCTCTCCATCTGCCT			
*PPARG*	F	GGATTCAGCTGGTCGATATCAC	NM_138711	120	Hs.PT.58.25464465
	R	GTTTCAGAAATGCCTTGCAGT			
*LPL*	F	CCTTGGAACTGCACCTGTAG	NM_000237	148	Hs.PT.58.45792913
	R	GAGAAGCTATCCGCGTGA			
*CHAD*	F	TGTTTCAGCGTGGTTACAC	NM_001267	109	Hs.PT.58.40907038
	R	ACAATGCCTTCCAGTCCTTT			
*ACAN*	F	AGATTCACAGAACTCCAGTGC	NM_001135(2)	105	Hs.PT.56a.742783
	R	ACCTACGATGTCTACTGCTTTG			
*Col2A1*	F	GTTTTCCAGCTTCACCATCATC	NM_033150	121	Hs.PT.58.4107778
	R	CCTCAAGGATTTCAAGGCAAT			
*ACTA2*	F	CACGAAGCTCATTGTAGAAAGAG	NM_001141945	115	Hs.PT.56a.39662523
	R	GCACAGAGCAAAAGAGGAATC			
*TUBB3*	F	CCTCCGTGTAGTGACCCTT	NM_001197181	89	Hs.PT.58.20385221
	R	GGCCTTTGGACATCTCTTCAG			
*CD24*	F	CAATGTCAAATCCAAAGCCTCA	NM_013230	130	Hs.PT.58.45758278.g
	R	CTCAACGTATTGTTTCGACAGC			
*CD56 (NCAM1)*	F	CCGTCATCCTGCTTGATCAG	NM_181351	110	Hs.PT.58.3970990
	R	GAGTTCAAGACGCAGCCA			
*LRRC17*	F	CAAGTCTTCTAATACGCCATAGTCA	NM_005824	136	Hs.PT.58.615298
	R	TGAGATGAAACCCTGCAAGTAG			
*KCNK12*	F	AGGTTGAAGAACAGGATGGTC	NM_022055	130	Hs.PT.58.21235707
	R	TACTTCGTGGGCACCGT			
*HAPLN1*	F	CAGATTGAAATCAGCACCAGAAG	NM_001884	143	Hs.PT.58.3994973
	R	CGCTAGCTTCACTTGATCTCC			
*SUSD2*	F	GCATGATGGAGACCCTGTC	NM_019601	89	Hs.PT.58.1294267
	R	GGAGGTGCTGAGCTTCAC			
*PTPRE*	F	CTCCCAGACCATTCTCCAGA	NM_130435	103	Hs.PT.58.40307003
	R	CAATGCTTCCTACATAGATGGTTAC			
*FBLN2*	F	CCAGGCACTCGTCATTGTC	NM_001998	115	Hs.PT.58.38361266
	R	CCAACTCTGTCCATTCTATCCC			
*DLL1*	F	GTCACAAAATCCATGCTGCTC	NM_005618	96	Hs.PT.58.41063402
	R	GTGGGGAGAAAGTGTGCAA			
*ACTB*	F	CCTTGCACATGCCGGAG	NM_001101	110	Hs.PT.39a.22214847
	R	ACAGAGCCTCGCCTTTG			

Abbreviations: ACAN, Aggrecan; ACTA2, Actin Alpha 2; ACTB, Actin Beta; CHAD, Chondroadherin; Col2A1, Collagen Type II Alpha 1; DLL1, Delta Like Canonical Notch Ligand 1; FBLN2, Fibulin 2; GAPDH, Glyceraldehyde‐3‐Phosphate Dehydrogenase; HAPLN1, Hyaluronan And Proteoglycan Link Protein 1; KCNK12, Potassium Two Pore Domain Channel Subfamily K Member 12; LPL, Lipoprotein Lipase; LRRC17, Leucine Rich Repeat Containing 17; MSX1, Msh Homeobox 1; NCAM, Neural Cell Adhesion Molecule 1; NES, Nestin; PPARG, Peroxisome Proliferator Activated Receptor Gamma; PTPRE, Protein Tyrosine Phosphatase Receptor Type E; RUNX2, RUNX Family Transcription Factor 2; SANI2, Snail Family Transcriptional Repressor 2; SNAI1, Snail Family Transcriptional Repressor 1; SUSD2, Sushi Domain Containing 2; TUBB3, Tubulin Beta 3.

### 2.12 Differentiation of sphere‐forming APDCs, PDLDCs, and OMSDCs

For mineralized cell differentiation, the cells were cultured in mesenchymal stem cell (MSC) osteogenic differentiation medium (ready‐to‐use; PromoCell, Heidelberg, Germany) supplemented with 100 ng/ml bone morphogenic protein 2 (BMP‐2; PeproTech). For adipogenic differentiation, the cells were cultured in adipogenic differentiation medium, (αMEM supplemented with 10% FBS and 500 mM 3‐isobutyl‐1‐methylxanthine [Sigma‐Aldrich, St. Louis, MO], 1 μM dexamethasone [Sigma‐Aldrich], 0.01 mg/ml insulin [Sigma‐Aldrich], and 0.2 mM indomethacin [Sigma‐Aldrich]). For chondrogenic differentiation, enzymatically dissociated cells (2.0 × 10^5^ cells/tube) were maintained in MSC chondrogenic differentiation medium (ready‐to‐use; PromoCell) using a pellet culture.^5^, ^18^, ^23^ For myogenic differentiation, spheres were prepared as described above and then cultured in high‐glucose DMEM supplemented with 10% FBS and 10 ng/ml TGF‐β1 (PeproTech).^17^, ^18^, ^23^ For neural differentiation, cells were cultured in MSC neurogenic differentiation medium (ready‐to‐use; PromoCell).

### 2.13 In vivo hard/mineralized tissue‐forming ability

Sphere‐forming APDCs, PDLDCs, and OMSDCs were cultured in αMEM supplemented with 10% FBS. Enzymatically dissociated cells were seeded into porous hydroxyapatite (HA) scaffolds (Hoya Technologies, Tokyo, Japan). These scaffolds were implanted into subcutaneous pouches in the dorsum of five‐week‐old male BALB/cAJcl nude mice (CLEA Japan, Tokyo, Japan) after mineralization‐induced cell differentiation as described above.^6^, ^17^, ^18^, ^23^ After 12 weeks, the implanted tissues were removed and prepared for histological analysis as described previously.^6^, ^17^, ^18^

### 2.14 Scanning electron micrograph (SEM) analysis

SEM analysis was performed as previously described.^6^, ^23^

### 2.15 Statistical analysis

Mean values were compared using one‐way analysis of variance with post hoc Tukey's multiple comparison test. *p* < 0.05 was considered significant. All statistical analyses were performed using EZR (Saitama Medical Center, Jichi Medical University, Shimotsuke, Japan), a graphical user interface for R (version 2.13.0, R Foundation for Statistical Computing, Vienna, Austria).

## 3 RESULTS

### 3.1 Clinical and histological features of human apical papilla, periodontal ligament, and oral mucosa tissue

**FIGURE 1:**
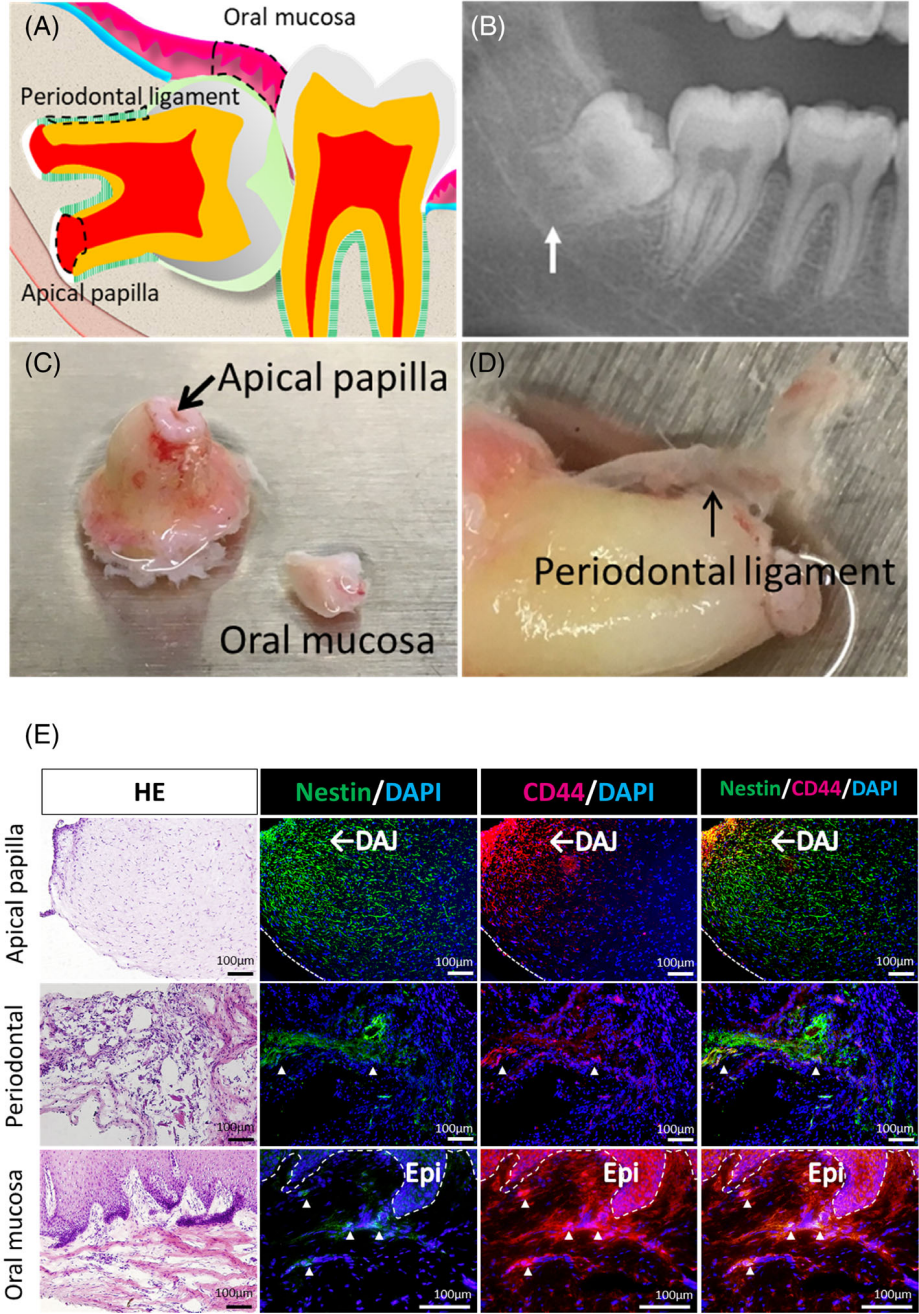
Characterization of human apical papilla, periodontal ligament, and oral mucosal tissue. (A) Schema showing impacted developing third molar. (B) Panoramic XP of the human impacted third molar with immature root apex. (C, D) Photographs of human apical papilla (C), periodontal ligament (D), and oral mucosa (C) tissue. (E) HE staining, and immunohistochemical analysis of human AP, PDL, and OM tissues. Abbreviations: DAPI, 4′,6‐diamidino‐2‐phenylindole; DAJ, dentin‐apical pulp junction; Epi, epithelium; HE, Hematoxylin–eosin staining

The developing impacted third molar had an immature apex (Figure 1A, B). The apical papilla was the soft tissue of the apical tip of immature roots (Figure 1A, C), whereas the periodontal ligament was the thin, soft tissue between the cementum and alveolar bone (Figure 1A, D). The oral mucosal covering of the oral cavity obtained during molar extraction was the gingiva or alveolar mucosa (Figure 1A, C). To investigate the candidate niche of NCSCs, tissue sections were stained for nestin and CD44.^23^ Double‐positive cells were observed in the dentin‐apical pulp junction of the apical papilla, some areas of the periodontal ligament, and the lamina propria of the oral mucosa (Figure 1E).

### 3.2 Culturing of APDCs, PDLDCs, and OMSDCs

**FIGURE 2:**
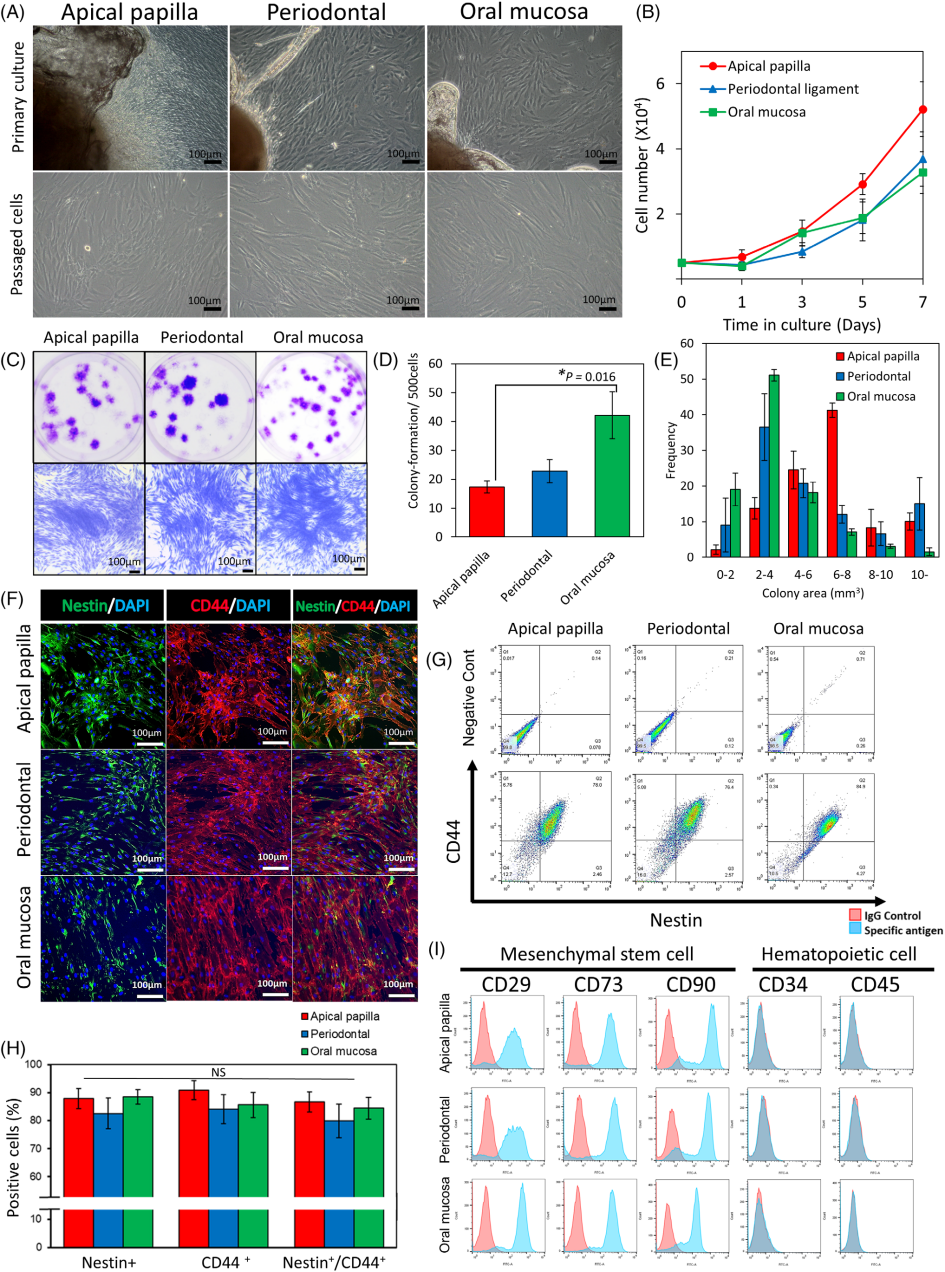
Characterization of human APDCs, PDLDCs, and OMSDCs. (A) Morphology of cultured APDCs, PDLDCs, and OMSDCs *in vitro*. Upper panel: primary cultured cells around the explanted tissue; lower panel: exponential growth of passaged cells in monolayer culture. (B) Cell growth curves of APDCs, PDLDCs, and OMSDCS (*n* = 5, patient‐matched). (C) Photographs of colony‐forming unit fibroblasts from APDCs, PDLDCs, and OMSDCs at 14 days. (D) Colony‐formation assays for APDCs, PDLDCs, and OMSDCs (*n* = 5, patient‐matched). Colonies stained with crystal violet. (E) Measurement of colony areas of APDCs, PDLDCs, and OMSDCs (*n* = 4, patient‐matched). (F) Immunohistochemistry for nestin and CD44. (G) Flow cytometry analysis of the expression of nestin and CD44. (H) Percent expression of nestin, CD44, and nestin/CD44 in cells (*n* = 3, patient‐matched). (I) Expression of surface markers of APDCs, PDLDCs, and OMSDCs. Average data are expressed as mean ± standard error (SE). Abbreviations: DAPI, 4′,6‐diamidino‐2‐phenylindole. *p < 0.05

Primary cells were cultured using the outgrowth culture system.^5^, ^6^, ^17^, ^18^, ^23^ Fibroblastic cells were observed, and the differences in morphological features were not observed between the tissues (Figure 2A). The APDCs, PDLDCs, and OMSDCs grew rapidly in an *in vitro* monolayer culture (Figure 2A). No significant difference was observed in the growth curves of cells derived from each tissue on each day (Figure 2B). APDCs and OMSDCs differed significantly in their colony‐forming abilities (*p =* 0.016; Figure 2C, D). However, APDCs tended to form large colonies, whereas OMSDCs formed small colonies (Figure 2E).

### 3.3 Expression of NCSC and MSC markers in APDCs, PDLDCs, and OMSDCs

Expanded APDCs, PDLDCs, and OMSDCs were positive for nestin and CD44 (Figure 2F, G). The expression of these markers did not vary significantly among the cell types (Figure 2H). Furthermore, these cells were positive for nearly all MSC markers (CD29, CD73, and CD90); however, they were negative for CD34 and CD45, indicating that they were not of hematopoietic stem/progenitor cell origin (Figure 2I).

### 3.4 Characteristics of spheres derived from APDCs, PDLDCs, and OMSDCs

**FIGURE 3:**
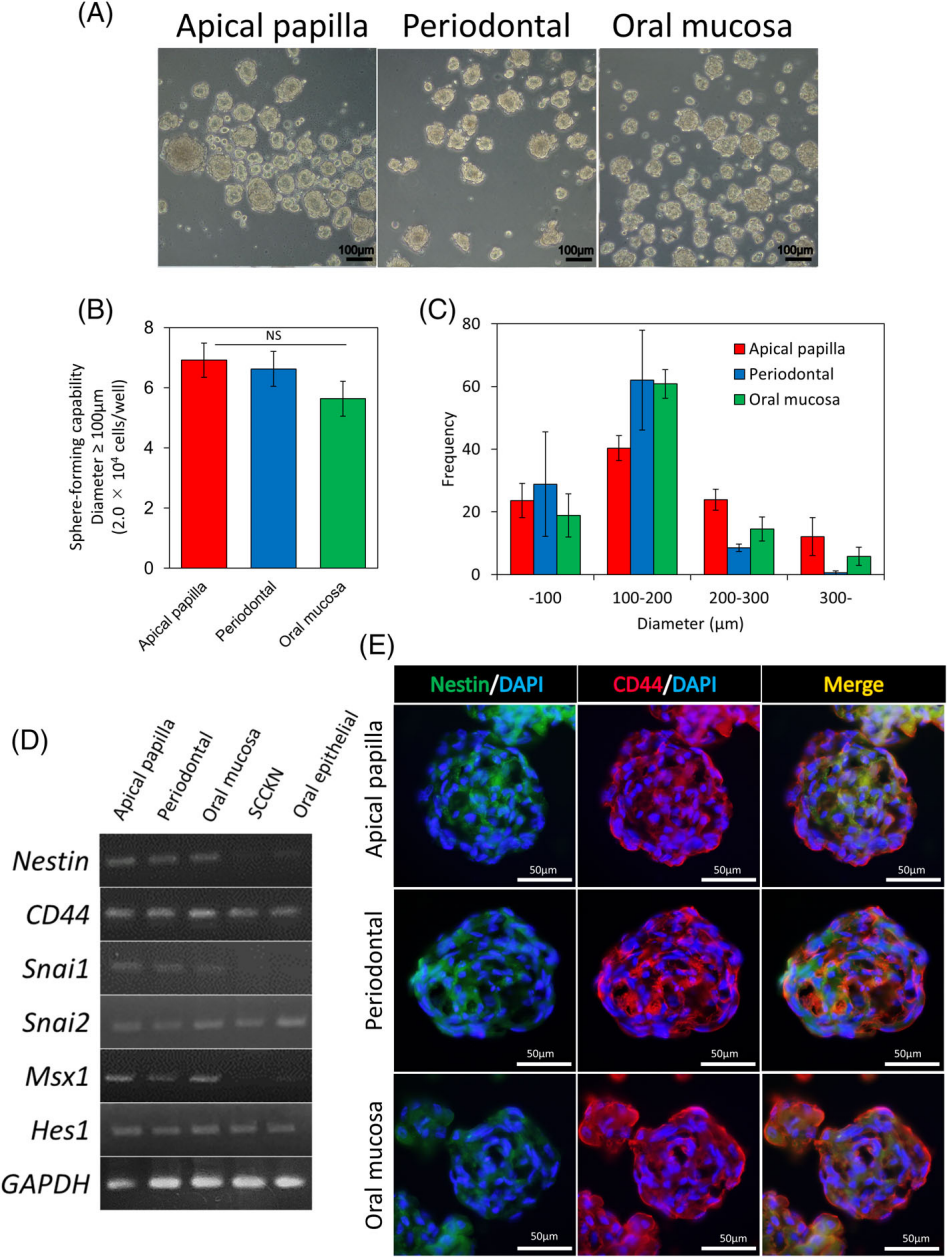
Isolation and characterization of sphere‐forming APDCs, PDLDCs, and OMSDCs. (A) APDCs, PDLDCs, and OMSDCs formed spheres (5.0 × 10^4^ cells/well). (B) Sphere‐forming abilities (*n* = 6, patient‐matched). Spheres with diameter ≥ 100 μm were counted after seven days of sphere culture (2.0 × 10^4^ cells/well, *n* = 6, patient‐matched). (C) Measurement of sphere diameter (*n* = 3, patient‐matched). (D) Semi‐quantitative RT‐PCR analysis of *NES*, *CD44*, *SNAI1*, *SNAI2*, *MSX1*, and *HES1* mRNAs. *GAPDH*, used as the internal control. (E) Immunohistochemical analysis of nestin and CD44. Results are expressed as mean ± SE. Abbreviations: DAPI, 4′,6‐diamidino‐2‐phenylindole

To enrich NCSC‐like cells, APDCs, PDLDCs, and OMSDCs were cultured using the sphere technique (Figure 3A). Sphere‐forming ability did not differ significantly between cells derived from different tissues (Figure 3B). Measurement of sphere diameter revealed the same tendency in each tissue, and spheres with a diameter of 100–200 μm were the most common (Figure 3C). The isolated sphere‐forming APDCs, PDLDCs, and OMSDCs expressed neural stem cell (NSC)‐ and NCSC‐specific markers, such as *NES*, *CD44*, *SNAI1*, *SNAI2*, *MSX1*, and *HES1*, as shown by semi‐quantitative RT‐PCR (Figure 3D). SCCKN and oral mucosal epithelial cells, which are not NC‐derived cells, showed expression of some markers; however, their expression patterns were different from those of APDCs, PDLDCs, and OMSDCs (Figure 3D). Furthermore, immunohistochemistry revealed that the isolated sphere‐forming APDCs, PDLDCs, and OMSDCs expressed nestin and CD44 (Figure 3E).

### 3.5 Transcriptome changes in sphere‐forming APDCs, PDLDCs, and OMSDCs

**FIGURE 4:**
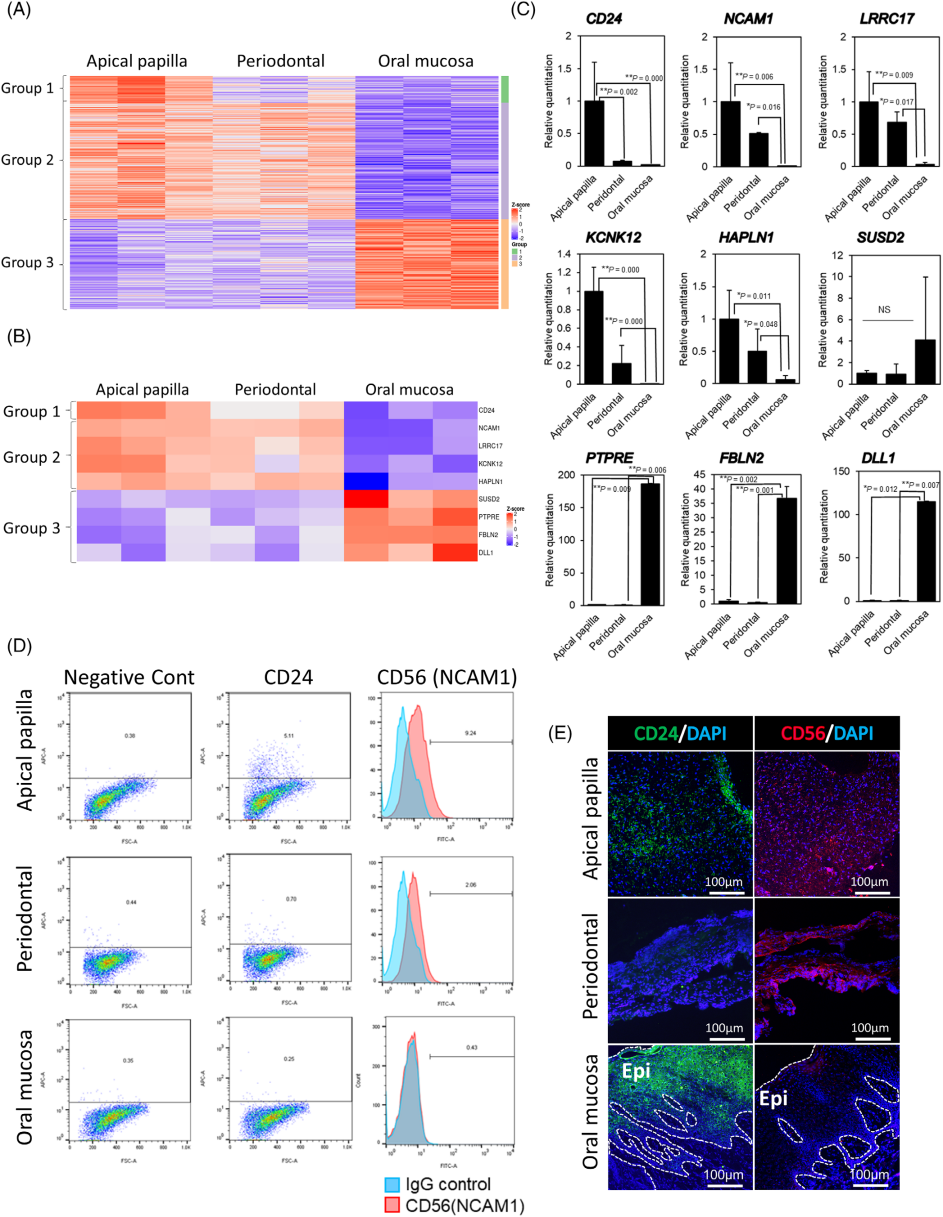
Differences in transcriptome and expression of cell surface proteins among sphere‐forming APDCs, PDLDCs, and OMSDCs. (A) Heatmap of significant differences in the transcriptome and expression among sphere‐forming APDCs, PDLDCs, and OMSDCs. Cutoff line: Fold change >2.0 and adjusted *p* < 0.05 (*n* = 3, patient‐matched). (B) Common differentially expressed genes identified using microarray analysis. (C) Quantitative RT‐PCR analysis showing differences in the expression levels of genes selected using microarray analysis in APDCs, PDLDCs, and OMSDCs (*n* = 4, patient‐matched). (D) Flow cytometric analyses of differentially expressed CD24 and CD56 (NCAM1). (E) Immunohistochemical analyses of differentially expressed CD24 and CD56 (NCAM1) in human oral tissues. Abbreviations: DAPI, 4′,6‐diamidino‐2‐phenylindole. *p < 0.05, **p < 0.01

To characterize the gene expression profiles of sphere‐forming cells, microarray analysis was performed, and the expression profiles of sphere‐forming APDCs, PDLDCs, and OMSDCs were compared. Significantly differentially expressed genes (DEGs) were also identified in these cells and clustered into three groups (Figure 4A). Group 1 contained 37 genes highly enriched in sphere‐forming APDCs, group 2 contained 160 genes highly enriched in sphere‐forming APDCs and PDLDCs, and group 3 contained 123 genes enriched in sphere‐forming OMSDCs. Only two genes were more enriched in PDLDCs than in APDCs and OMSDCs. Among the significant DEGs, *CD24* (group 1); *CD56* (*NCAM1*), *LRRC17*, *KCNK12*, and *HAPLN1* (group 2); and *SUSD2*, *PTPRE*, *FBLN2*, and *DLL1* (group 3) were identified. These genes were selected from the top 10 common genes with significantly different expression levels according to the data when compared between groups (Figure 4B, Table S1). The quantitative RT‐PCR results confirmed the microarray analysis results (Figure 4C). Only *SUSD2* expression was upregulated in the oral mucosa of all samples, although not significantly (Figure 4C). CD24 expression was observed in 2.93% ± 1.10% of APDCs but not in PDLDCs or OMSDCs (Figure 4D). CD56 (NCAM1) expression was observed in 9.59% ± 2.16% of APDCs and 3.98% ± 0.69% of PDLDCs but not in OMSDCs (Figure 4D). Surprisingly, immunohistochemical analyses of CD24 and CD56 (NCAM1) in human tissues revealed that CD24 was expressed in the apical papilla, whereas CD56 (NCAM1) was expressed in the apical papilla and periodontal ligament. In the oral mucosa, the expression of CD24 was observed only in the oral epithelium, whereas that of CD24 and CD56 (NCAM1) was not detected in the lamina propria (Figure 4E). These results indicated that the expression patterns of CD24 and CD56 (NCAM1) varied with tissues and that they may serve as candidate markers for tissue specificity.

### 3.6 Differentiation into NC lineage cells

**FIGURE 5:**
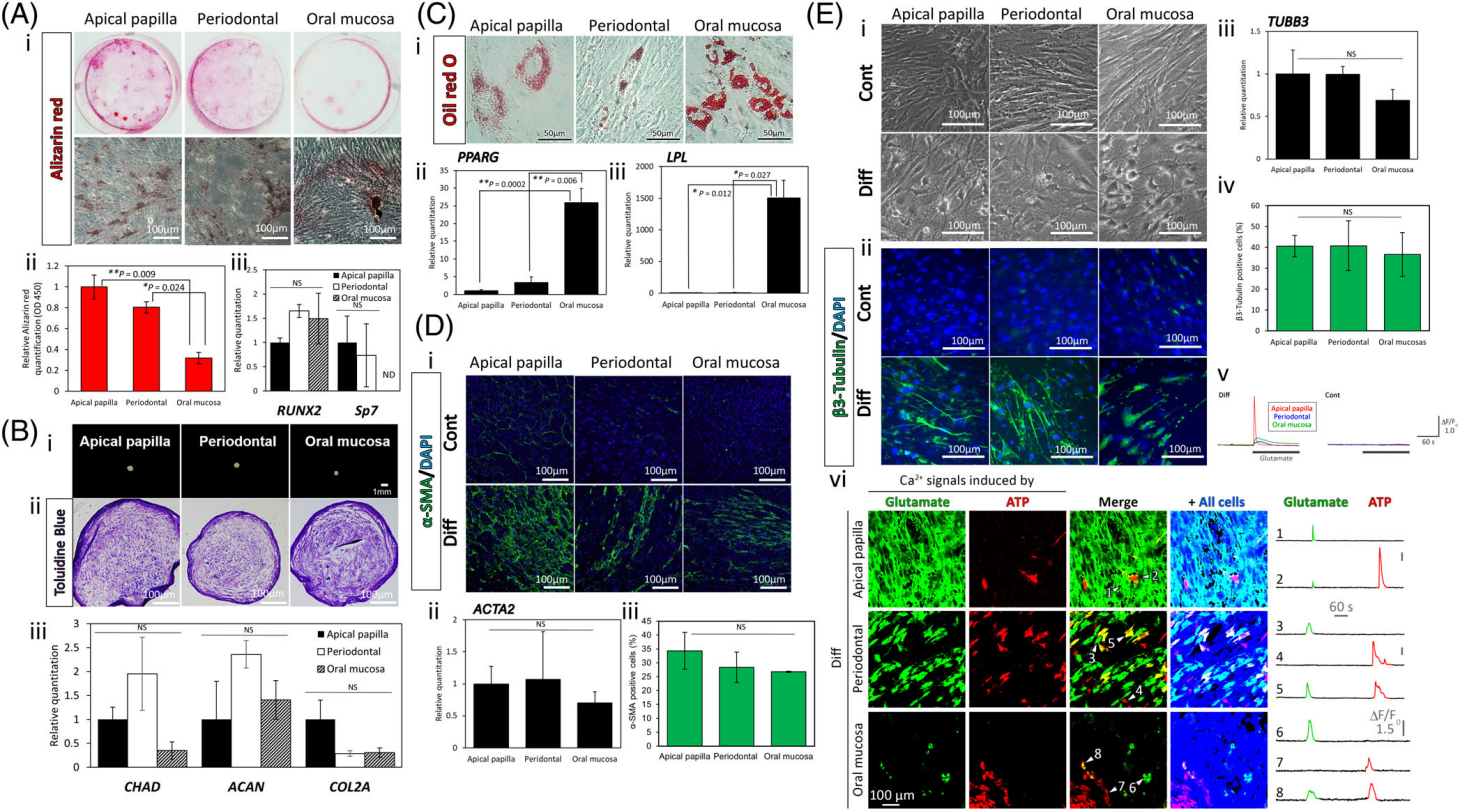
Differentiation capacity of sphere‐forming APDCs, PDLDCs, and OMSDCs to neural crest lineage cells. Identification of differentiation using (A) Alizarin Red S staining for mineralized cells, (B) toluidine blue staining for chondrocytes, (C) Oil Red O staining for adipocytes, (D) α‐SMA immunostaining for myocytes, and (E) β3‐tubulin immunostaining for neurons. (a[i]) Images of Alizarin Red‐stained differentiated cells (patient‐matched). (a[ii]) The relative Alizarin Red quantification (OD 450 nm) following culture under mineralized‐cell differentiation conditions for 3 weeks (*n* = 3, patient‐matched). (a[iii]) Quantitative RT‐PCR analysis of *RUNX2* and *SP7* (*n* = 4, patient‐matched). (b[i]) Photographs of micromass pellets cultured under chondrogenic conditions for three weeks after staining with toluidine blue. (b[ii]) Quantitative RT‐PCR analysis of *CHAD*, *ACAN*, and *COL2A* (b[iii]) (*n* = 3, patient‐matched). (c[i]) Microscopic images of Oil Red O‐stained adipocytes (patient‐matched) after culturing under adipogenic conditions for 3 weeks. (c[ii, iii]) Quantitative RT‐PCR analysis of *PPARG* (c[ii]) and *LPL* (c[iii]) (*n* = 4, patient‐matched). (d[i]) Microscopic images showing α‐SMA staining after culture in conditioned or myogenic differentiation medium for seven days. (d[ii]) Quantitative RT‐PCR analysis of *ACTA2* (*n* = 4, patient‐matched). (d[iii]) Immunohistochemical analysis of α‐SMA (*n* = 3, patient‐matched). (e[i]) Microscopic images after culture in conditioned or neural‐like cell differentiation medium for seven days. (e[ii]) Immunohistochemical analysis of β3‐tubulin. (e[iii]) Quantitative RT‐PCR analysis of *TUBB3* (*n* = 4, patient‐matched). (e[iv]) Immunohistochemical analysis of β3‐tubulin (*n* = 3, patient‐matched). (e[v]) Time‐lapse imaging of intracellular Ca^2+^ levels in the sphere‐forming APDCs, PDLDCs, and OMSDCs with and without differentiation. Glutamate (30 μM) was extracellularly applied to examine whether functional glutamate receptor signaling pathways are present in the cells. (e[vi]) Cell populations showed agonist‐induced Ca^2+^ signals and typical traces of individual cellular Ca^2+^ response. Image panels are maximum projected Ca^2+^ response to glutamate and subsequently applied ATP (30 μM). The rightmost images (“+ All cells”) indicate total cell shape in each optical field by overlaying time‐summation images of Ca^2+^ indicator fluorescence. Individual Ca^2+^ dynamics of the cells indicated in “Merge” images are shown in the right traces. Average data are expressed as mean ± SE. Abbreviations: Cont., control group; DAPI, 4′,6‐diamidino‐2‐phenylindole; Diff., differentiation group. *p < 0.05, **p < 0.01

Sphere‐forming APDCs, PDLDCs, and OMSDCs exhibited the multipotency of NCSCs (Figure 5), as they differentiated into NC lineage cells under appropriate culture conditions. In mineralized cell differentiation, the Alizarin Red‐stained areas in APDCs and PDLDCs were larger than those in OMSDCs (Figure 5A[i]). These data were confirmed by the quantification of Alizarin Red (*p* = 0.009 and *p* = 0.024, respectively, Figure 5A[ii]). Furthermore, the quantification of the expression of mineralized‐cell differentiation markers revealed that *RUNX2*, a master gene regulating mineralization, was expressed in APDCs, PDLDCs, and OMSDCs (Figure 5A[iii]), suggesting that these cells differentiated into mineralized progenitor cells. However, *SP7*, a late mineralized‐cell differentiation marker, was detected in APDCs and PDLSCs, but not in OMSDCs (Figure 5A[iii]). In chondrogenic differentiation, these pellets were stained with toluidine blue (Figure 5B [i, ii]). The expression of chondrogenesis‐related markers (*CHAD*, *ACAN*, and *COL2A*) was confirmed in APDCs, PDLDCs, and OMSDCs (Figure 5B[iii]), and did not vary significantly between tissues. After adipogenic differentiation, Oil Red O‐positive adipocytes were observed in all cells (Figure 5C[i]), although the number of differentiated adipocytes was higher in OMSDCs than in APDCs and PDLDCs. The expression of adipogenesis‐related markers (*PPARG* and *LPL*) was significantly upregulated in OMSDCs compared with that in APDCs and PDLDCs (*PPARG*: *p* = 0.0002 and 0.006, respectively; *LPL*: *p =* 0.012 and 0.027, respectively; Figure 5C[ii, iii]). In smooth muscle cell differentiation, all tissue‐derived cells differentiated into α‐SMA‐positive smooth muscle cells (Figure 5D[i]). *ACTA2* (mRNA) and α‐SMA (protein) expression were confirmed in all cells (Figure 5D[ii, iii]) and found to not vary significantly between tissues. During neuronal differentiation, all tissue‐derived cells could be recognized as multipolar morphological cells (Figure 5E[i]) and differentiated into β3‐tubulin‐positive neural‐like cells (Figure 5E[ii]). *TUBB3* (mRNA) and β3‐tubulin (protein) expression were confirmed in all cells (Figure 5E[iii, iv]) and did not vary significantly between tissues. Furthermore, to evaluate function of neural‐like cells, we performed intracellular calcium (Ca^2+^) imaging. We found that all kinds of cell cultures after differentiation contain cells showing a marked increase in Ca^2+^ concentration in response to extracellular administration of glutamate, a neurotransmitter, whereas there were no glutamate‐induced Ca^2+^ responses in undifferentiated control cells (Figure 5E[v]). All three differentiated cultures contain glutamate‐responsive cells without showing Ca^2+^ response to subsequently applied extracellular Adenosine tri‐phosphate (ATP) (Figure 5E[vi]; cells 1, 3, and 6), indicating higher potent degree of neuronal differentiation. These results indicate that the sphere‐forming cell‐derived neural‐like cells have neuronal features. The *in vitro* differentiation assay indicated that the sphere‐forming cells contained NCSCs. APDCs and PDLDCs readily differentiated into mineralized cells; however, they differentiated into adipocytes less readily. Moreover, OMSDCs exhibited opposite differentiation trends.

### 3.7 Generation of hard tissue in vivo

**FIGURE 6:**
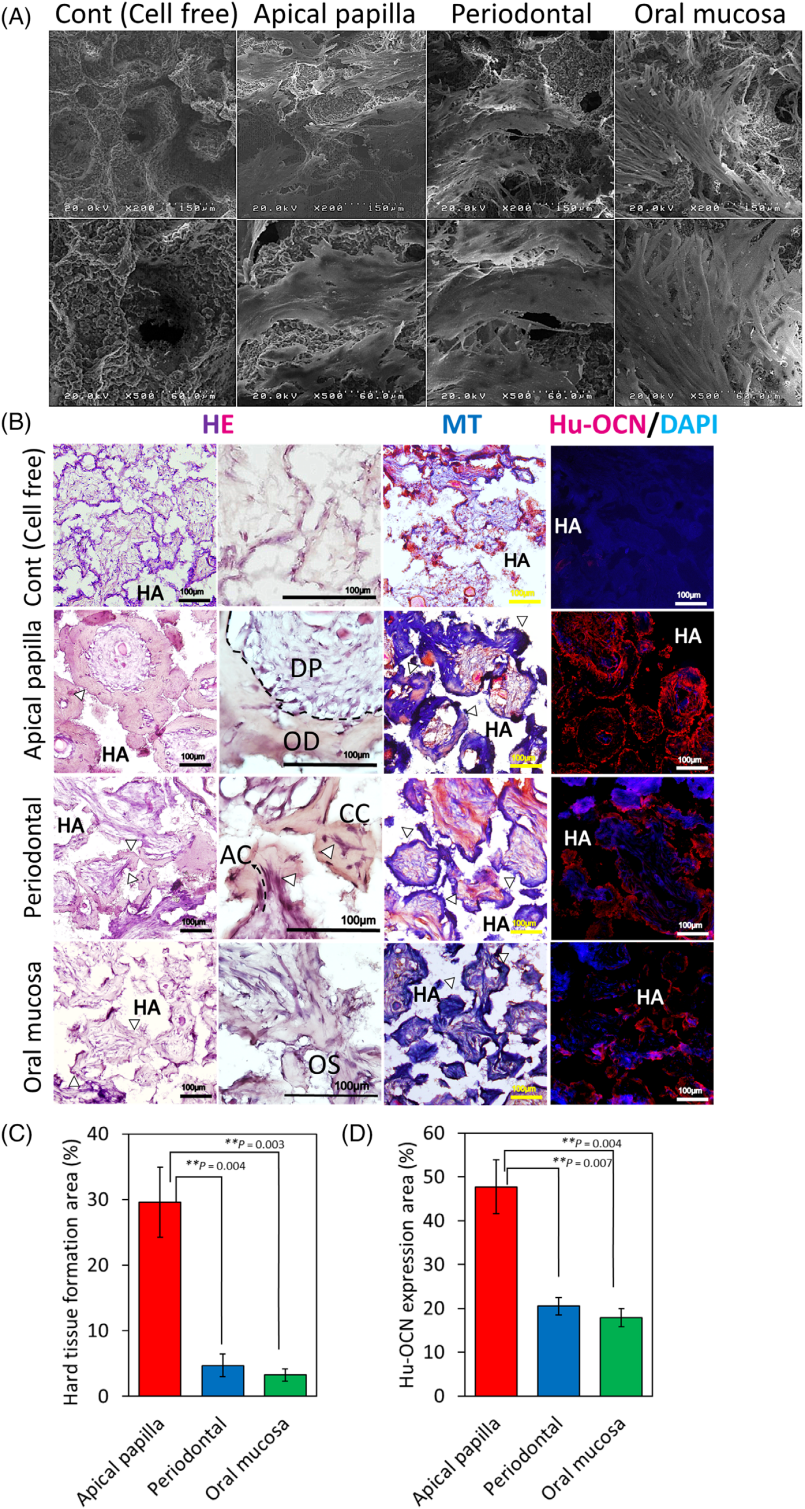
*In vivo* hard tissue‐forming ability of APDCs, PDLDCS, and OMSDCs. (A) SEM images of cells adhered to porous hydroxyapatite scaffolds. (B) HE staining, Masson's trichrome staining, and immunohistochemical analysis of transplanted tissues. HE staining revealed hard/mineralized‐tissue forming ability *in vivo*. The generated hard tissues were dental pulp‐like and osteodentin‐like structures in APDCs, thin cellular cementum‐like or acellular cementum‐like tissues with Sharpey's fiber‐like structures (arrow head) in PDLDCs, and thin immature osteoid‐like tissues in OMSDCs. Masson's trichrome staining revealed that regenerated hard tissues were composed of collagen fibers. Immunofluorescence staining was performed for human osteocalcin. (C) Quantitative analysis of the area with hard tissue formation (*n* = 3, patient‐matched). (D) Quantitative analysis of human osteocalcin area (*n* = 3, patient‐matched). Average data are expressed as mean ± SE. Abbreviations: Cont., control group; DAPI, 4′,6‐diamidino‐2‐phenylindole; HA, hydroxyapatite; HE, hematoxylin–eosin staining; Hu‐OCN, human osteocalcin; MT, Masson's trichrome staining. Arrowhead: hard tissue. **p < 0.01

The crucial factor for clinical application of stem cells from oral tissues is hard tissue regeneration. Hence, we investigated the regeneration capacity of sphere‐forming APDCs, PDLDCs, and OMSDCs related to the formation of hard tissue *in vivo* (Figure 6). To investigate cell adhesion to the multiporous HA scaffolds, the sphere‐forming cells cultured on the scaffolds were examined by SEM 10 days after seeding (Figure 6A). The sphere‐forming cells from all tissues exhibited vigorous cell sheet‐like growth in the multiporous scaffold (Figure 6A). Twelve weeks after implantation, APDCs, PDLDCs, and OMSDCs had formed ectopic mature or immature hard tissue (Figure 6B). The regenerated hard tissues from APDCs were osteodentin‐like, tissues from PDLDCs were thin and cementum‐like, and those from OMSDCs were thin, immature, and osteoid‐like (Figure 6B). Masson's trichrome staining revealed that these regenerated hard tissues comprised collagen fibers (Figure 6B). The generated hard/mineralized‐tissue stained positively for anti‐human OCN (Figure 6B). These data suggested that the regenerated tissue was hard tissue. Subsequently, the ability to form hard tissue was quantified using the hard tissue area stained with H&E (Figure 6C) and the OCN‐positive area (Figure 6D). APDCs exhibited a significantly larger area of hard tissue formation (*p* = 0.004, *p =* 0.003 respectively) and OCN expression (*p =* 0.007, *p =* 0.004 respectively) than PDLDCs and OMSDCs (Figure 6C, D).

## 4 DISCUSSION

To date, specific markers for the localization of NCSCs in human tissues have not been identified. We previously reported that nestin^+^/CD44^+^ cells are localized in the lamina propria of the oral mucosa.^23^ Nestin is a widely used NSC and stem cell marker in the dental pulp (including apical papilla),^3^, ^5^, ^17^, ^18^, ^19^ periodontal ligament,^22^ oral mucosa/gingiva,^9^, ^11^, ^23^, ^24^ skin,^12^, ^13^, ^15^, ^16^ heart,^15^ and bone marrow.^16^ Previous reports have suggested that nestin‐positive bone marrow cells are enriched multipotent MSCs with sphere‐forming and self‐renewing abilities.^29^, ^30^, ^31^ Although nestin is expressed in NSCs, Liu *et al*. reported that human embryonic stem cell‐derived NCSCs are nestin^+^/CD44^+^, whereas NSCs are nestin^+^/CD44^−^.^32^ Therefore, both nestin and CD44 were utilized to identify NCSCs; a nestin^+^/CD44^+^ population was observed among the cells derived from each tissue. Because most cells were nestin^+^/CD44^+^ (approximately 80%–87%), they may have been derived from NCs.

The sphere culture system has been widely used for the enrichment of NSCs and other types of stem cells.^33^, ^34^ Many reports have suggested that NCSCs, MSCs, and cancer stem cells possess sphere‐forming abilities.^13^, ^14^, ^15^, ^16^, ^17^, ^18^, ^19^, ^20^, ^21^, ^22^, ^23^, ^24^, ^30^, ^31^, ^34^ Previously, we have reported the NCSC properties of APDCs and OMSDCs using this system.^19^, ^20^ Furthermore, sphere‐forming APDCs, PDLDCs, and OMSDCs express *NES*, *CD44*, *SNAI1*, *SNAI2*, *MSX1*, and *HES1* which were NC‐associated genes.^23^, ^35^ These data suggest that NC‐derived cells or NCSCs are present in sphere‐forming APDCs, PDLDCs, and OMSDCs.

We detected genes with different expression levels among distinct tissue‐derived cells. CD24 has been reported as a marker of several normal tissue‐derived stem/progenitor cells and cancer stem cells.^20^ These stem/progenitor cells exhibit higher CD24 expression than terminally differentiated cells, revealing the close relationship between CD24 expression, cellular pluripotency, and self‐renewal capability.^20^ This phenomenon has been reported in previous studies on tooth development. For example, Sonoyama *et al*. reported that CD24 is a specific marker of the apical papilla, whereas it is not detected in DPSCs and bone marrow MSCs, and that mineralized cells differentiated from APDCs do not express CD24.^4^ Chen *et al*. recently reported that CD24 expression increases by 72.9% during sphere formation in mouse APDCs.^20^ However, in our study, the expression of CD24 in sphere‐forming human APDCs was only 2.9% (Figure 4). Similarly, Sonoyama *et al*. reported an average of 7.56% expression in CD24‐positive cells.^4^ Hence, further studies regarding the expression of CD24 in APDCs are required. CD56 (NCAM1) is a marker of natural killer cells, neural cells, muscles, and MSCs generated from human embryonic stem cells.^36^, ^37^ However, Buttula *et al*. detected adipocyte differentiation in colonies derived from MSCA‐1^+^/CD56^−^ MSCs but not in those derived from MSCA‐1^+^/CD56^+^ MSCs.^37^ This was consistent with the finding that CD56^−^ OMSDCs exhibited significantly higher adipocyte differentiation than CD56^+^ APDCs and PDLDCs.

HAPLN1 regulates cell growth in the developing cartilage and heart valves, and is expressed in mesoderm‐committed embryonic stem cells, cancer cells undergoing epithelial‐mesenchymal transition (EMT), and metastatic melanomas.^38^ Mabarki *et al*. reported that HAPLN1 is expressed *de novo* in EPCAM1^−^/CD56 (NCAM1)^+^ mesoderm‐committed progenitor cells and fibroblastic hepatocellular carcinoma cells, and during the dedifferentiation of hepatocyte‐like cells to liver progenitors. Silencing of *HAPLN1* downregulates the markers of EMT.^38^ Because sphere‐forming APDCs and PDLDCs expressed HAPLN1 and CD56, these markers may be related to MSCs, cells undergoing EMT, and NCSCs (Figure 4D–F). Kim *et al*. reported that LRRC17, a member of the LRR superfamily, acts as a negative regulator of RANKL‐induced osteoclast differentiation and is highly expressed in osteoblasts.^39^ This is consistent with our finding of high LRRC17 expression levels in APDCs and PDLDCs, which tended to differentiate more into mineralized cells *in vitro* than did OMSDCs (Figure 5A). Although it was difficult to identify stem cell‐specific markers based only on the characteristic markers identified in this study, we successfully determined the specific markers for each tissue and tissue‐derived cell.

Human stem/progenitor cells derived from APDCs, PDLDCs, and OMSDCs can differentiate into NC lineage cells *in vitro*.^1^, ^2^, ^3^, ^4^, ^5^, ^6^, ^7^, ^8^, ^9^, ^17^, ^18^, ^19^, ^20^, ^21^, ^22^, ^23^, ^24^ However, whether the multipotency of each tissue‐derived stem/progenitor cell type is equivalent was unclear. In this study, we evaluated these cells from the same individual and observed that the *in vitro* differentiation abilities of APDCs and PDLDCs were similar, and that the mineralized cell differentiation abilities of these cells were high, although their adipogenic ability was low (Figure 5A, C). OMSDCs showed the opposite tendency (Figure 5A, C). Monterubbianes *et al*. reported that GMSCs have a higher adipogenic differentiation ability than DPSCs, and that DPSCs exhibit a higher osteogenic potential than GMSCs.^40^ A comparative study of abilities for differentiation into smooth muscle cells and neural‐like cells in these cells is lacking. Our results regarding mineralized cell and adipocyte differentiation abilities were similar to those reported by Monterubbianes *et al*. However, we did not observe any difference in chondrocyte differentiation between tissues. Previous studies isolated these stem/progenitor cells through monolayer culture, whereas we used enriched stem/progenitor cells isolated using the sphere culture technique in this study. We believe that differentiation abilities may differ with the stem/progenitor cell isolation technique.

The *in vivo* hard tissue regenerative capacity of APDCs, PDLDCs, and OMSDCs has been reported previously.^1^, ^2^, ^3^, ^4^, ^5^, ^6^, ^7^, ^8^, ^9^, ^17^, ^18^, ^19^, ^20^, ^21^, ^22^, ^23^ Although human APDCs retained osteodentin‐like hard tissue‐forming abilities, PDLDCs and OMSDCs had negligible hard tissue‐forming ability (Figure 6). Surprisingly, PDLDCs showed different hard tissue‐forming abilities in *in vitro* and *in vivo* transplantation experiments. Grzesik *et al*. reported that hard tissue is not formed upon transplantation of PDLDCs, although these cells form calcified nodules and express mineralized‐cell differentiation markers when cultured in mineralized‐cell differentiation medium *in vitro*.^41^ Seo *et al*. reported that, unlike DPSCs, PDLSCs have the potential to generate cementum/PDL‐like tissue, in which hard tissue is thinly formed around HA *in vivo*.^7^ Grzesik *et al*. suggested that differentiated mineralized cells are lost in the early stage of differentiation and that the remaining cells do not form hard tissue *in vivo*; alternatively, the differentiated mineralized cells may be negatively regulated by other fibroblastic populations that exert a suppressive influence *in vivo*.^41^, ^42^ In the present study, APDCs, PDLDCs, and OMSDCs were cultured in BMP‐2‐supplemented osteogenic differentiation medium. Hence, the mineralized cells differentiated from PDLDCs may require BMP‐2 and might have formed hard tissue only when it was supplied as a stimulus.^41^ The difference in the ability of each tissue‐derived cell to form hard tissue *in vivo* is closely associated with biological roles. For example, APDCs have a high ability to form hard tissues, such as tooth roots. In contrast, in the periodontal ligament, the ability to form cementum remains unaltered under application of occlusal force. In addition, ectopic bone formation in the oral mucosa is extremely rare.

Previous reports have suggested that human “NCSC‐like cells” are defined based on their sphere‐forming capacity, expression of NCSC‐related markers, and *in vitro* multipotential phenotype. Recently, Chan *et al*. identified the skeletal stem cells from human adult bone that also have a hierarchy and have the highest self‐renewal and differentiation potential, even in cells isolated according to the conventional MSCs definition.^43^, ^44^ It is suggested that there may be common neural crest stem cells with high stem cell property in each tissue used in this study.

This study, however, showed that the differentiation potential and expression of markers differed depending on the tissue from which the cells were obtained, suggesting that these tissue‐specific characteristics should be considered during application in regenerative medicine.^13^, ^18^, ^21^, ^23^, ^24^, ^35^ In the future, these distinct tissue‐specific markers may play a crucial role in tooth regenerative medicine as they can clearly distinguish each tissue in the tooth organoids generated using induced pluripotent stem cells and tissue‐derived stem/progenitor cells.

## 5 CONCLUSIONS

We demonstrated that multilineage sphere‐forming APDCs, PDLDCs, and OMSDCs share the same phenotypes as other stem/progenitor cells, although expression of certain tissue‐specific markers and differentiating abilities vary based on tissue source. We showed for the first time that APDCs, PDLDCs, and OMSDCs obtained from the same patients and, concomitantly, the same sites, can be used individually in regenerative medicine‐based therapy. These differences in the differentiation capacity between each type of tissue‐derived cells should be taken into consideration when administering stem cell‐based therapy in clinical settings. In addition, we identified human tissue‐specific markers in the currently unknown human developing tooth with immature apex. Our study identified important tissue‐specific markers that distinguish between apical papilla, periodontal ligament, and oral mucosa in developing stage of human third molar, as well as serves as a basis for future regenerative medicine research.

## AUTHOR CONTRIBUTIONS

SA contributed to study conception and design, methodology, investigation, data analysis and interpretation, and manuscript writing. AK, KK, and KN contributed to study conception and design, methodology, investigation, and data analysis and interpretation. NY and YK contributed to study conception and design, methodology, and investigation. MM, TM, CH, HK, and TY contributed to study conception and design and methodology. All authors read and approved the final manuscript.

## CONFLICT OF INTEREST

The authors declare that they have no competing interests.

## Supporting information


Appendix S1

Table S1

## Data Availability

Data sharing is not applicable to this article as no new data were created or analyzed in this study.
