# Supplementary material for: Differences in the stemness characteristics and molecular markers of distinct human oral tissue neural crest‐derived multilineage cells
Source: Cell Prolif. 2022 Jun 18;55(10):e13286. doi: 10.1111/cpr.13286 (PMC9528771; doi:10.1111/cpr.13286)
Supplement: Supplementary file 1 — Appendix S1 [file CPR-55-e13286-s002.docx]

**Extended Materials and Methods**

***Patients***

This study was approved by the Institutional Review Board of Tokyo Metropolitan Hiroo Hospital (expedited approval numbers: 2019-15 and 2020-11), Faculty of Dentistry of the Tokyo Medical and Dental University (approval number: D2017-036), and Faculty of Medicine of the Nihon University (approval number: P20-19-0). Twenty-four patients, aged 12 to 22 years, underwent routine surgery for the extraction of impacted developing third molars that had formed approximately more than one-third of their root at the Oral Surgery Department of the Tokyo Metropolitan Hiroo Hospital and Nihon University School of Medicine. During this procedure, human apical papilla, periodontal ligament, and oral mucosa tissues were obtained and used in this study. Written informed consent was obtained from all donors.

***Histological analysis***

Human apical papilla, periodontal ligament, and oral mucosa tissues from 11 randomly selected patients were prepared for analysis. Histological analysis was performed as described previously.^23^ The sections were then sliced and used for hematoxylin-eosin and immunohistochemical staining for nestin, CD44, CD24, and CD56 (NCAM1). Samples from seven and four patients were used to perform immunohistochemistry for nestin and CD44 and for CD24 and CD56 (NCAM1), respectively.

***Cell culture***

For primary cell culture, apical papilla, periodontal ligament, and oral mucosa tissues were cut into 2–3 mm pieces, each of which was cultured in Isocove’s modified Dulbecco’s medium (Nacalai Tesque, Kyoto, Japan) supplemented with 10% fetal bovine serum (FBS) (Gibco Life Technologies, Carlsbad, CA, USA, Lot number: 42A0158K). APDCs, PDLDCs, and OMSDCs were passaged and seeded at a density of 2.0 × 10^4^ cells/well in a 6-well culture plate.^1-5^ Cryopreserved cells from the second to fifth passages were used for each experiment. For oral mucosal epithelial cells, oral mucosa tissues were cut into 2–3 mm pieces, each of which was cultured in keratinocyte SFM (Gibco Life Technologies) as described above, and the second passage was used in the experiment. The SCCKN which is human tongue squamous carcinoma cell line was cultured in 45% Dulbecco’s modified Eagle medium (DMEM) (Life Technologies), 45% RPMI 1640 medium (Gibco Life Technologies), and 10% FBS.^6^

***Growth of APDCs, PDLDCs, and OMSDCs***

APDCs, PDLDCs, and OMSDCs from five patients were seeded at a density of 5,000 cells/well in 96-well plates. Viability assays were performed 1, 3, 5, and 7 days following culture using a Cell Counting Kit-8 (Dojindo, Tokyo, Japan) to determine the number of viable cells.^5^

***Colony-forming assay***

APDCs, PDLDCs, and OMSDCs from five patients were seeded at a density of 500 cells/well in 6-well plates. After 14 days of culture, the cells were stained with crystal violet. The diameters of colonies comprising at least 50 cells were measured. Experiments were performed in triplicate for each sample. Colony areas were measured using ImageJ (National Institutes of Health, Bethesda, MD, USA).

***Sphere culture***

APDCs, PDLDCs, and OMSDCs (2.0 or 5.0 × 10^4^ cells/well) were cultured in 24-well super-hydrophilic plates (Cellseed, Tokyo, Japan) in serum-free DMEM/F12 (1:1) containing N_2_ supplements (Gibco Life Technologies), 20 ng/mL basic fibroblast growth factor (PeproTech, Rocky Hill, NJ, USA), and 20 ng/mL epidermal growth factor (PeproTech) for seven days. ^3-5^ Primary spheres were used for all experiments. To analyze sphere-forming ability and measure sphere diameter, APDCs, PDLDCs, and OMSDCs (2.0 × 10^4^ cells/well) were cultured in 24-well super-hydrophilic plates in sphere culture conditions for seven days. ^3-5^ Spheres with a diameter ≥ 100 µm were counted, and the diameter of all spheres was measured using ImageJ. Experiments were performed in triplicate for each sample.

***Immunohistochemistry***

Immunohistochemistry was performed as previously described.^5^ Tissues and cells were fixed with 4% paraformaldehyde (PFA; Wako Pure Chemical, Osaka, Japan) at 4 °C and washed twice with tris-buffered saline with Tween^®^20 (TBST). Cells and cryosections were incubated in Blocking One Histo solution (Nacalai Tesque, Kyoto, Japan) for 10 min to prevent non-specific binding of the antibodies. The slides were incubated at room temperature for 1 h with antibodies specific for nestin (10C2; eBioscience, San Diego, CA; 1:50), CD44 (IM7; eBioscience; 1:100), CD24 (ML5; BD Pharmingen, San Jose, CA; 1:50), CD56 (B159; BD Pharmingen; 1:50), α-smooth muscle actin (α-SMA; 1A4; R&D Systems; 1:200), β3-tubulin (2G10; eBioscience; 1:100), or human osteocalcin (OCN; 5-12H; Takara; 1:100). The cells were washed twice with phosphate-buffered saline (PBS) and incubated at room temperature for 30 min with anti-mouse IgG conjugated with Alexa Fluor 488 (Invitrogen, Carlsbad, CA, USA; 1:200) and anti-mouse or anti-rat IgG conjugated Alexa 594 (Invitrogen; 1:200). For quantitative analysis, the percentage of positively stained cells was determined by counting the number of cells in randomly selected fields or sections.

***Flow cytometry***

For cell surface antigen phenotyping, sphere-forming APDCs, PDLDCs, and OMSDCs from three patients were enzymatically dissociated and washed with PBS. To assess the expression of nestin, cells were fixed with 4% PFA and permeabilized with ice-cold methanol. The dissociated cells were incubated at room temperature for 1 h with antibodies specific for nestin (10C2; eBioscience; 1:50), CD24 (ML5; BD Pharmingen, San Jose, CA, USA; 1:50), CD29 (MAR4; BD Pharmingen; 1:100), CD34 (581; BD Pharmingen; 1:100), CD44 (IM7; eBioscience; 1:100), CD45 (HI30; BD Pharmingen; 1:100), CD56 (B159; BD Pharmingen; 1:50), CD73 (AD2; BD Pharmingen; 1:100), and CD90 (5E10; BioLegend, San Diego, CA; 1:100). After incubation with the primary antibodies, the cells were washed with PBS, and then incubated with anti-mouse or anti-rat IgG conjugated with Alexa 488 or 594 (Invitrogen; 1:200) at room temperature for 30 min. Subsequently, the cells were analyzed with a FACSCant (BD Biosciences, Franklin Lakes, NJ, USA) using FlowJo software.

*2.9 RNA extraction*

Total RNA was extracted from the cells using the RNeasy Mini Kit (Qiagen, Hilden, Germany), according to manufacturer instructions.

***Microarray***

The total RNA of sphere-forming APDCs, PDLCs, and OMSCs from three patients was used for the microarray analysis. Total RNA (100 ng) was reverse-transcribed into cDNA using the Low Input Amp Labeling Kit (Agilent Technologies, Santa Clara, CA, USA). Next, cDNA was mixed with a hybridization buffer and hybridized to a SurePrint G3 Human GE microarray 8X60k v3 (Agilent Technologies) for 17 h, following manufacturer instructions. The chips were washed and subsequently scanned using the Agilent microarray scanner (Agilent Technologies). Raw data were analyzed using the feature extraction software (v.11.5.1.1; Agilent Technologies) and microarray data were mined and analyzed using GeneSpring (Agilent Technologies). The signal intensity was normalized by adjusting the data to a 75^th^ percentile baseline (GEO accession number for the microarray gene expression data: GSE164327).

***Semi-quantitative and quantitative reverse transcription PCR (RT-PCR)***

Semi-quantitative RT-PCR was performed using the PrimeScript One-Step RT-PCR Kit Ver.2 (Takara) and 10 ng of total RNA. cDNA synthesis and pre-denaturation were performed for one cycle at 50 °C for 30 min and 94 °C for 2 min. Following initial denaturation, amplification was performed over 35 cycles of 94 °C for 30 s, 56–58 °C for 30 s, and 72 °C for 1 min in a SimpliAmp thermal cycler (Applied Biosystems, Life Technologies). Quantitative RT-PCR was performed using the One-Step PrimeScript RT-PCR Kit (Perfect Real Time; Takara). cDNA synthesis and pre-denaturation were performed over one cycle of 42 °C for 5 min and 95 °C for 10 s. After initial denaturation, amplification was performed over 40 cycles of 95 °C for 5 s and 60 °C for 31 s in an Applied Biosystems 7300 real-time PCR system (Applied Biosystems, Life Technologies). The cycle threshold values (Ct values) were used to calculate the fold differences using the 2^-ΔΔCt^ method. The primer sequences used for RT-PCR are listed in Table 1.^5,7,8^

***Differentiation of sphere-forming APDCs, PDLDCs, and OMSDCs***

For mineralized cell differentiation, sphere-forming APDCs, PDLDCs, and OMSDCs were seeded in 24-well plates and cultured for seven days in α-minimum essential medium (αMEM) supplemented with 10% FBS. Subsequently, the medium was replaced with mesenchymal stem cell (MSC) osteogenic differentiation medium (ready-to-use; PromoCell, Heidelberg, Germany) supplemented with 100 ng/mL bone morphogenic protein 2 (BMP-2; PeproTech), and the cells were further cultured for up to three weeks. To identify the mineralized matrix, cells were stained with Alizarin Red S (Wako Pure Chemical). For quantification of Alizarin red S-stained cells, the stained cells were lysed in 5% formic acid (Kanto Chemical, Tokyo, Japan) and stirred at room temperature for 10 mins. The cell lysates were then collected and quantified using plate reader at absorbance 450 nm using iMark (Bio-Rad Laboratories, Hercules, CA, USA). For adipogenic differentiation, spheres were cultured as described above. The medium was then replaced with αMEM supplemented with 10% FBS and 500 mM 3-isobutyl-1-methylxanthine (Sigma-Aldrich, St. Louis, MO), 1 µM dexamethasone (Sigma-Aldrich), 0.01 mg/mL insulin (Sigma-Aldrich), and 0.2 mM indomethacin (Sigma-Aldrich), and the spheres were incubated for up to three weeks. Cells were stained with Oil Red O (Sigma-Aldrich) to detect adipocyte differentiation. For chondrogenic differentiation, the spheres were cultured as described above, and the enzymatically dissociated cells (2.0 × 10^5^ cells/tube) were maintained in MSC chondrogenic differentiation medium (ready-to-use; PromoCell) using a pellet culture for three weeks, as described previously.^1,4,5^ Toluidine blue (Muto Pure Chemical, Tokyo, Japan) staining was performed to identify chondrogenic cells. For myogenic differentiation, spheres were cultured as described above, then cultured in high-glucose DMEM supplemented with 10% FBS and 10 ng/mL TGF-β1 (PeproTech) for 10 days.^3-5^ To identify smooth muscle cells, cells were immunostained with anti-α-SMA antibody. For neural differentiation, after the spheres were cultured as described above with IMDM supplemented with 10% FBS, the medium was replaced with MSC neurogenic differentiation medium (ready-to-use; PromoCell), and the spheres were further cultured for one week. To identify neural cells, immunostaining with anti-β3-tubulin antibody was conducted. For intracellular Ca^2+^ imaging in neural-like cells from sphere-forming APDCs, PDLDCs, and OMSDCs, we used Cal-520 (AAT Bioquest, Sunnyvale, CA, USA) .^9.10^ The spheres were cultured as described above, and the enzymatically dissociated cells (Control: 1.0 × 10^4^ cells/well, Differentiation: 5.0 × 10^4^ cells/well, 8 wells chamber slide coated by Poly-D-Lysine/Laminin (Corning, NY, USA)) were maintained in IMDM supplemented with 10% FBS for 3 days. Then, the medium was replaced with MSC neurogenic differentiation medium (ready-to-use; PromoCell), and the cells were further cultured for one week. After differentiated neural-like cells were treated with 5 µM Cal-520 at room temperature for 30 min in HEPES-buffered saline (150 mM NaCl, 4 mM KCl, 2 mM CaCl_2_, 1 mM MgCl_2_, 5 mM HEPES, 5.6 mM glucose) (pH 7.4). ^9,10^ Fluorescence was recorded before and after exposure to 30 µM L-glutamate (Nacalai tesque, Kyoto, Japan) and 30 µM Adenosine tri-phosphate (Sigma-Aldrich). Fluorescence images were acquired at 510–550 nm using a TCS SP8 confocal microscope system (Leica, Wetzlar, Germany) equipped with a × 20 PL APO objective (NA = 0.40; Leica) at a rate of one frame per 1s with the 488-nm excitation laser. Data analyses were carried out using ImageJ (National Institutes of Health, Bethesda, MD, USA). ^9,10^

***In vivo hard/mineralized tissue-forming ability***

All experimental procedures were approved by and conducted in accordance with the Animal Care and Committee of the Tokyo Medical and Dental University (approval number: A2019-313A). Sphere-forming APDCs, PDLDCs, and OMSDCs were cultured for seven days in αMEM supplemented with 10% FBS. Enzymatically dissociated cells were seeded at a density of 2.0 × 10^5^ cells into porous hydroxyapatite (HA) scaffolds that were half in size (Hoya Technologies, Tokyo, Japan; 85%, pore diameter: 100−500 μm, diameter: 5 mm, and thickness: 2 mm). The cells were transplanted into immunocompromised mice 10 days after mineralization-induced cell differentiation according to the method described above (n = 3, patient-matched).^2-5^ Briefly, cells with HA scaffolds were implanted into subcutaneous pouches in the dorsum of five-week-old male BALB/cAJcl nude mice (CLEA Japan, Tokyo, Japan). After 12 weeks, the implanted tissues were removed and prepared for histological analysis as described previously.^2,3,4^

**Supplemental reference**

1. Abe S, Yamaguchi S, Amagasa T. Multilineage cells from apical pulp of human tooth with immature apex. *Oral Sci Int* 2007: 4:45–58.
2. Abe S, Yamaguchi S, Watanabe A, Hamada K, Amagasa T. Hard tissue regeneration capacity of apical pulp derived cells (APDCs) from human tooth with immature apex. *Biochem Biophys Res Commun* 2008: 371:90–3.
3. Abe S, Hamada K, Yamaguchi S, Amagasa T, Miura M. Characterization of the radioresponse of human apical papilla-derived cells. *Stem Cell Res Ther* 2011: 2.
4. Abe S, Hamada K, Miura M, Yamaguchi S. Neural crest stem cell property of apical pulp cells derived from human developing tooth*. Cell Biol Int* 2012: 36:927–36.
5. Abe S, Yamaguchi S, Sato Y, Harada K. Sphere-derived multipotent progenitor cells obtained from human oral mucosa are enriched in neural crest cells. *Stem Cells Transl Med* 2016: 5:117–28.
6. Kitano H, Masaoka Y, Mamiya A, Fujiwara Y, Miki T, Hidai C. Combination Cancer Therapy of a Del1 Fragment and Cisplatin Enhanced Therapeutic Efficiency *In Vivo. In Vivo* 2021: 35: 779–791.
7. Xia Y, Nivet E, Sancho-Martinez I, Gallegos T, Suzuki K, Okamura D, et al. Directed differentiation of human pluripotent cells to ureteric bud kidney progenitor-like cells. *Nat Cell Biol* 2013: 15:1507–15.
8. Ali H, Al-Yatama MK, Abu-Farha M, Behbehani K, Al Madhoun A. Multi-lineage differentiation of human umbilical cord Wharton's Jelly Mesenchymal Stromal Cells mediates changes in the expression profile of stemness markers. *PLoS One* 2015: 10:e0122465.
9. Kanemaru K, Suzuki J, Taiko I, Iino M. Red fluorescent CEPIA indicators for visualization of Ca^2+^ dynamics in mitochondria. Sci Rep 2020: 10:2835.
10. Mikami Y, Kanemaru K, Okubo Y, Nakaue T, Suzuki J, Shibata K, et al. Nitric Oxide-induced Activation of the Type 1 Ryanodine Receptor Is Critical for Epileptic Seizure-induced Neuronal Cell Death. *EBioMedicine* 2016: 11; 253–61.
