# Supplementary material for: Differences in the stemness characteristics and molecular markers of distinct human oral tissue neural crest‐derived multilineage cells
Source: Cell Prolif. 2022 Jun 18;55(10):e13286. doi: 10.1111/cpr.13286 (PMC9528771; doi:10.1111/cpr.13286)
Supplement: Supplementary file 2 — Table S1 [file CPR-55-e13286-s001.docx]

**Supplemental Table 1: Expression levels of different genes as determined via microarray analysis**

| **Probe name** | **Gene symbol** | **Gene name** | **Fold change  (log)** | **P-value** | **GenBank** |
| --- | --- | --- | --- | --- | --- |
| Periodontal ligament/Apical papilla | | | | | |
| **Upregulated** | | | | | |
| x 2.0 in PP & T Test | | | | | |
| A_23_P161439 | *ADIRF* | adipogenesis regulatory factor | 1.77 | 0.025 | NM_006829 |
| A_23_P44569 | *ABCC2* | ATP-binding cassette, sub-family C (CFTR/MRP), member 2 | 1.68 | 0.039 | NM_000392 |
| A_24_P140475 | *SORBS2* | sorbin and SH3 domain containing 2 | 1.62 | 0.026 | NM_021069 |
| A_21_P0000645 | *APCDD1L-AS1* | APCDD1L antisense RNA 1 (head to head) | 1.44 | 0.026 | NR_034147 |
| A_23_P138706 | *ADRA2A* | adrenoceptor alpha 2A | 1.44 | 0.040 | NM_000681 |
| A_23_P121795 | *SORBS2* | sorbin and SH3 domain containing 2 | 1.41 | 0.040 | NM_021069 |
| A_33_P3718274 | *LOC285629* | uncharacterized LOC285629 | 1.39 | 0.041 | DB296219 |
| A_21_P0002020 |  |  | 1.38 | 0.002 |  |
| A_21_P0012151 | *APCDD1L-AS1* | APCDD1L antisense RNA 1 (head to head) | 1.36 | 0.027 |  |
| A_23_P350001 | *GUCY1A2* | guanylate cyclase 1, soluble, alpha 2 | 1.36 | 0.002 | NM_000855 |
| x 2.0 in AP & T Test | |  |  |  |  |
| A_33_P3337574 | *FGF14-IT1* | FGF14 intronic transcript 1 (non-protein coding) | 1.55 | 0.018 | NR_036486 |
| A_19_P00809372 | *XLOC_l2_008203* |  | 1.53 | 0.046 |  |
| A_22_P00023561 |  |  | 1.36 | 0.030 |  |
| A_22_P00023133 | *lnc-C11orf55-1* | lnc-C11orf55-1:2 | 1.21 | 0.040 |  |
| **Downregulated** | | | | | |
| x 0.5 in PP & T Test | | | | | |
| A_33_P3259135 | *NSG1* | neuron specific gene family member 1 | -4.34 | 0.017 | NM_001287763 |
| A_33_P3369844 | *CD24* | CD24 molecule | -4.04 | 0.018 | NM_013230 |
| A_33_P3218980 | *ENTPD1* | ectonucleoside triphosphate diphosphohydrolase 1 | -4.02 | 0.048 | NM_001776 |
| A_23_P125435 | *GABRB1* | gamma-aminobutyric acid (GABA) A receptor, beta 1 | -3.97 | 0.034 | NM_000812 |
| A_23_P81103 | *SFRP2* | secreted frizzled-related protein 2 | -3.94 | 0.045 | NM_003013 |
| A_33_P3733417 | *DRD2* | dopamine receptor D2 | -3.54 | 0.043 | NM_000795 |
| A_33_P3287158 | *NLGN4X* | neuroligin 4, X-linked | -3.00 | 0.011 | NM_001282145 |
| A_23_P77993 | *C1QL1* | complement component 1, q subcomponent-like 1 | -2.98 | 0.032 | AF095154 |
| A_23_P73632 | *NR0B1* | nuclear receptor subfamily 0, group B, member 1 | -2.84 | 0.049 | NM_000475 |
| A_23_P103511 | *C1orf226* | chromosome 1 open reading frame 226 | -2.80 | 0.021 | NM_001085375 |
| x 0.5 in PA & T Test | |  |  |  |  |
| A_32_P73151 | *lnc-GABRB1-1* | lnc-GABRB1-1:1 | -4.24 | 0.025 | AK124778 |
| A_23_P18447 | *PPARGC1A* | peroxisome proliferator-activated receptor gamma, coactivator 1 alpha | -3.79 | 0.035 | NM_013261 |
| A_33_P3218975 | *ENTPD1* | ectonucleoside triphosphate diphosphohydrolase 1 | -2.97 | 0.032 | NM_001776 |
| A_22_P00020401 |  |  | -2.97 | 0.007 |  |
| A_23_P121441 | *NLGN4Y* | neuroligin 4, Y-linked | -2.92 | 0.005 | NM_014893 |
| A_33_P3274129 | *TMEM151B* | transmembrane protein 151B | -2.88 | 0.039 | NM_001137560 |
| A_24_P93948 | *KIF5A* | kinesin family member 5A | -2.40 | 0.039 | AB210045 |
| A_33_P3533325 | *ASXL3* | additional sex combs like transcriptional regulator 3 | -2.39 | 0.045 | XM_005258356 |
| A_33_P3332414 | *ABCB1* | ATP-binding cassette, sub-family B (MDR/TAP), member 1 | -2.33 | 0.041 | NM_000927 |
| A_23_P127915 | *STK33* | serine/threonine kinase 33 | -2.31 | 0.044 | NM_030906 |
| Oral mucosa/Apical papilla | | | | | |
| **Upregulated** | | | | | |
| x 2.0 in PP & T Test | | | | | |
| A_33_P3287223 | *DPP4* | dipeptidyl-peptidase 4 | 6.82 | 0.009 | NM_001935 |
| A_23_P143981 | *FBLN2* | fibulin 2 | 5.15 | 0.025 | NM_001004019 |
| A_23_P340848 | *PTGIR* | prostaglandin I2 (prostacyclin) receptor (IP) | 4.89 | 0.024 | NM_000960 |
| A_23_P314101 | *SUSD2* | sushi domain containing 2 | 4.87 | 0.037 | NM_019601 |
| A_23_P71037 | *IL6* | interleukin 6 | 4.86 | 0.021 | NM_000600 |
| A_33_P3347291 | *INMT* | indolethylamine N-methyltransferase | 4.62 | 0.005 | NM_001199219 |
| A_24_P926960 | *MEGF6* | multiple EGF-like-domains 6 | 4.60 | 0.005 | NM_001409 |
| A_23_P324754 | *CEMIP* | cell migration inducing protein, hyaluronan binding | 4.59 | 0.049 | NM_018689 |
| A_23_P167920 | *DLL1* | delta-like 1 (Drosophila) | 4.28 | 0.031 | NM_005618 |
| A_33_P3309556 | *PTPRE* | protein tyrosine phosphatase, receptor type, E | 4.21 | 0.008 | NM_006504 |
| x 2.0 in AP & T Test | | | | | |
| A_23_P5903 | *SLCO4A1* | solute carrier organic anion transporter family, member 4A1 | 6.62 | 0.020 | NM_016354 |
| A_23_P41344 | *EREG* | epiregulin | 6.48 | 0.020 | NM_001432 |
| A_33_P3373259 | *CACNA2D3* | calcium channel, voltage-dependent, alpha 2/delta subunit 3 | 6.24 | 0.009 | NM_018398 |
| A_33_P3330911 | *BCAS1* | breast carcinoma amplified sequence 1 | 5.73 | 0.012 | NM_003657 |
| A_23_P202071 | *CELF2* | CUGBP, Elav-like family member 2 | 5.48 | 0.014 | NM_001025077 |
| A_33_P3232692 | *IL24* | interleukin 24 | 5.24 | 0.049 | NM_001185156 |
| A_22_P00010216 |  |  | 5.12 | 0.041 | BX119863 |
| A_23_P37410 | *CYP19A1* | cytochrome P450, family 19, subfamily A, polypeptide 1 | 5.10 | 0.015 | NM_031226 |
| A_24_P84396 | *CEMIP* | cell migration inducing protein, hyaluronan binding | 4.94 | 0.036 | NM_018689 |
| A_33_P3241269 | *CES1* | carboxylesterase 1 | 4.88 | 0.002 | NM_001025195 |
| **Downregulated** | | | | | |
| x 0.5 in PP & T Test | | | | | |
| A_23_P7727 | *HAPLN1* | hyaluronan and proteoglycan link protein 1 | -7.26 | 0.037 | NM_001884 |
| A_33_P3256510 | *KCNK12* | potassium channel, two pore domain subfamily K, member 12 | -6.76 | 0.003 | NM_022055 |
| A_23_P253958 | *LRRC17* | leucine rich repeat containing 17 | -6.72 | 0.003 | NM_005824 |
| A_23_P257649 | *RBP1* | retinol binding protein 1, cellular | -5.74 | 0.013 | NM_002899 |
| A_23_P301855 | *LSAMP* | limbic system-associated membrane protein | -5.48 | 0.024 | NM_002338 |
| A_24_P870620 | *PTN* | pleiotrophin | -5.44 | 0.020 | NM_002825 |
| A_33_P3326634 | *GPC3* | glypican 3 | -5.40 | 0.006 | NM_001164617 |
| A_32_P83845 | *HEY1* | hes-related family bHLH transcription factor with YRPW motif 1 | -5.03 | 0.000 | NM_001040708 |
| A_23_P18123 | *NLGN1* | neuroligin 1 | -4.83 | 0.041 | NM_014932 |
| A_23_P303833 | *SCN4B* | sodium channel, voltage gated, type IV beta subunit | -4.76 | 0.007 | NM_174934 |
| x 0.5 in PA & T Test | | | | | |
| A_21_P0011967 | *SFRP4* | secreted frizzled-related protein 4 | -10.35 | 0.003 | NM_003014 |
| A_23_P118571 | *SOST* | sclerostin | -9.34 | 0.001 | NM_025237 |
| A_33_P3369844 | *CD24* | CD24 molecule | -9.32 | 0.002 | NM_013230 |
| A_23_P4714 | *MIA* | melanoma inhibitory activity | -8.49 | 0.001 | NM_006533 |
| A_33_P3335177 | *SFRP4* | secreted frizzled-related protein 4 | -8.35 | 0.000 | NM_003014 |
| A_23_P125435 | *GABRB1* | gamma-aminobutyric acid (GABA) A receptor, beta 1 | -7.98 | 0.014 | NM_000812 |
| A_33_P3363799 | *NCAM1* | neural cell adhesion molecule 1 | -7.16 | 0.037 | NM_001242607 |
| A_33_P3242883 | *DLX6* | distal-less homeobox 6 | -7.06 | 0.002 | NM_005222 |
| A_33_P3315385 | *MPPED2* | metallophosphoesterase domain containing 2 | -7.02 | 0.013 | NM_001584 |
| A_32_P187571 | *SCN2B* | sodium channel, voltage gated, type II beta subunit | -6.98 | 0.004 | NM_004588 |
| Oral mucosa/Periodontal ligament | | | | | |
| **Upregulated** | | | | | |
| x 2.0 in PP & T Test | | | | | |
| A_23_P41344 | *EREG* | epiregulin | 5.60 | 0.031 | NM_001432 |
| A_23_P161698 | *MMP3* | matrix metallopeptidase 3 (stromelysin 1, progelatinase) | 5.46 | 0.049 | NM_002422 |
| A_23_P5903 | *SLCO4A1* | solute carrier organic anion transporter family, member 4A1 | 5.37 | 0.025 | NM_016354 |
| A_23_P314101 | *SUSD2* | sushi domain containing 2 | 4.92 | 0.038 | NM_019601 |
| A_23_P167920 | *DLL1* | delta-like 1 (Drosophila) | 4.13 | 0.032 | NM_005618 |
| A_23_P119943 | *IGFBP2* | insulin-like growth factor binding protein 2, 36 kDa | 4.06 | 0.014 | NM_000597 |
| A_23_P397248 | *CLCA2* | chloride channel accessory 2 | 3.98 | 0.010 | NM_006536 |
| A_23_P143981 | *FBLN2* | fibulin 2 | 3.91 | 0.022 | NM_001004019 |
| A_33_P3309556 | *PTPRE* | protein tyrosine phosphatase, receptor type, E | 3.89 | 0.003 | NM_006504 |
| A_23_P142075 | *ACP5* | acid phosphatase 5, tartrate resistant | 3.79 | 0.049 | NM_001611 |
| x 2.0 in AP & T Test | | | | | |
| A_24_P926960 | *MEGF6* | multiple EGF-like-domains 6 | 5.58 | 0.012 | NM_001409 |
| A_33_P3373259 | *CACNA2D3* | calcium channel, voltage-dependent, alpha 2/delta subunit 3 | 5.32 | 0.026 | NM_018398 |
| A_33_P3398316 | *MMP27* | matrix metallopeptidase 27 | 5.13 | 0.018 | NM_022122 |
| A_33_P3241269 | *CES1* | carboxylesterase 1 | 4.87 | 0.004 | NM_001025195 |
| A_23_P434809 | *S100A8* | S100 calcium binding protein A8 | 4.70 | 0.007 | NM_002964 |
| A_23_P502336 | *EMR2* | egf-like module containing, mucin-like, hormone receptor-like 2 | 4.46 | 0.010 | NM_013447 |
| A_24_P158946 | *FGD4* | FYVE, RhoGEF and PH domain containing 4 | 4.44 | 0.031 | NM_139241 |
| A_33_P3361546 | *TFAP2A* | transcription factor AP-2 alpha (activating enhancer binding protein 2 alpha) | 4.18 | 0.047 | NM_001032280 |
| A_33_P3365750 | *EML5* | echinoderm microtubule associated protein like 5 | 3.99 | 0.025 | NM_183387 |
| A_21_P0000613 | *TFAP2A-AS1* | TFAP2A antisense RNA 1 | 3.91 | 0.049 | NR_033910 |
| **Downregulated** | | | | | |
| x 0.5 in PP & T Test | | | | | |
| A_23_P7727 | *HAPLN1* | hyaluronan and proteoglycan link protein 1 | -6.61 | 0.044 | NM_001884 |
| A_23_P7313 | *SPP1* | secreted phosphoprotein 1 | -5.77 | 0.022 | NM_001040058 |
| A_23_P253958 | *LRRC17* | leucine rich repeat containing 17 | -5.21 | 0.012 | NM_005824 |
| A_33_P3284129 | *LYPD1* | LY6/PLAUR domain containing 1 | -4.58 | 0.005 | NM_144586 |
| A_23_P102364 | *NGEF* | neuronal guanine nucleotide exchange factor | -4.53 | 0.024 | NM_019850 |
| A_32_P101031 | *LYPD1* | LY6/PLAUR domain containing 1 | -4.51 | 0.000 | NM_144586 |
| A_33_P3256510 | *KCNK12* | potassium channel, two pore domain subfamily K, member 12 | -4.38 | 0.015 | NM_022055 |
| A_19_P00319528 | *LOC100507420* | uncharacterized LOC100507420 | -4.07 | 0.011 | NR_121620 |
| A_22_P00013349 | *LOC100507420* | uncharacterized LOC100507420 | -4.01 | 0.013 | NR_121620 |
| A_23_P38537 | *KRT16* | keratin 16, type I | -4.00 | 0.016 | NM_005557 |
| x 0.5 in PA & T Test | | | | | |
| A_21_P0011967 | *SFRP4* | secreted frizzled-related protein 4 | -8.33 | 0.005 | NM_003014 |
| A_23_P118571 | *SOST* | sclerostin | -8.00 | 0.022 | NM_025237 |
| A_23_P144326 | *ASB5* | ankyrin repeat and SOCS box containing 5 | -7.09 | 0.022 | NM_080874 |
| A_33_P3363799 | *NCAM1* | neural cell adhesion molecule 1 | -7.02 | 0.031 | NM_001242607 |
| A_33_P3335177 | *SFRP4* | secreted frizzled-related protein 4 | -6.36 | 0.011 | NM_003014 |
| A_33_P3336257 | *IRX1* | iroquois homeobox 1 | -6.01 | 0.010 | NM_024337 |
| A_33_P3363804 | *NCAM1* | neural cell adhesion molecule 1 | -5.72 | 0.011 | NM_001242608 |
| A_23_P121657 | *HS3ST1* | heparan sulfate (glucosamine) 3-O-sulfotransferase 1 | -5.68 | 0.001 | NM_005114 |
| A_23_P209978 | *VSNL1* | visinin-like 1 | -5.67 | 0.011 | NM_003385 |
| A_21_P0013865 | *LOC102725299* | uncharacterized LOC102725299 | -5.46 | 0.013 |  |
